# Supplementary material for: A Bayesian mixture modelling approach for spatial proteomics
Source: PLoS Comput Biol. 2018 Nov 27;14(11):e1006516. doi: 10.1371/journal.pcbi.1006516 (PMC6258510; doi:10.1371/journal.pcbi.1006516)
Supplement: S1 Text — Figure A: Plot of the log-posterior at each iteration of the EM algorithm to demonstrate monotonicity and convergence. Figure B: Trace plots of the number of proteins allocated to the known components in each of 6 parallel MCMC runs. Chain 4 is discarded because of lack of convergence. 600 samples are retained from remaining chains and pooled. Figure C: Gene Ontology over representation analysis on outlier proteins—that is proteins allocated with less than probability 0.95. We analyse the enrichment of terms in the cellular compartment, biological process, and molecular function ontologies. We display the top 10 significant results in the dotplots. Figure D: A heatmap representation of a contingency table comparing allocation produced by MCMC and MAP methods with posterior probability threshold set at 0.99 for both methods. The scale ranges from 0 to 1 with values indicating the proportion of assigned proteins to that sub-cellular location. Values along the diagonal represent agreement between classifiers whilst other values represent disagreement. The allocations of proteins by both methods are in strong agreement. (PDF) [file pcbi.1006516.s001.pdf]

Supporting information for A Bayesian Mixture Modelling Approach For Spatial Proteomics

Oliver M. Crook[1,2,3], Claire M. Mulvey[2], Paul D.W. Kirk[3], Kathryn S. Lilley[2], and Laurent Gatto[1,2,4,*]

[1] *Computational Proteomics Unit, Department of Biochemistry, University of Cambridge, Cambridge, UK*
[2] *Cambridge Centre for Proteomics, Department of Biochemistry, University of Cambridge, Cambridge, UK*
[3] *MRC Biostatistics Unit, Cambridge Institute for Public Health, Cambridge, UK*
[4] *Current address: de Duve Institute, UCLouvain, Avenue Hippocrate 75, 1200 Brussels, Belgium*
[*] laurent.gatto@uclouvain.be

S1 Derivation of EM algorithm for TAGM model

This appendix give a formal derivation of the EM algorithm used for our model. Computations are standard but useful and similar technical summaries can be found (for example see Fraley and Raftery (2005); Murphy (2007)) We let $H = \{\boldsymbol{\mu}_0, \lambda_0, \nu_0, S_0\}$ denote the parameters of the normal-inverse-Wishart prior. More precisely:

$$\boldsymbol{\mu}_k, \Sigma_k \sim \mathcal{N}\left(\boldsymbol{\mu}_k | \boldsymbol{\mu}_0, \frac{\Sigma_k}{\lambda_0}\right) I\mathcal{W}\left(\Sigma_k | \nu_0, S_0\right). \tag{1}$$

Furthermore, let $\boldsymbol{\theta}_k = \{\boldsymbol{\mu}_k, \Sigma_k\}$, and let $\Theta = \{\kappa, \mathbf{M}, V\}$ be the parameters of the global $\mathcal{T}$ distribution. We specify the following hierarchical Bayesian model.

$$\begin{aligned}
\pi|\beta &\sim Dir(\beta), \\
\theta_k|H &\sim \mathcal{NIW}(H), \\
z_i|\pi &\sim cat(\pi), \\
\epsilon|u, v &\sim \mathcal{B}(u, v) \\
\phi_i|\epsilon &\sim Ber(1-\epsilon) \\
\mathbf{x}_i|z_i = k, \theta, \Phi, \Theta &\sim \mathcal{N}(\mathbf{x}_i|\boldsymbol{\mu}_k, \Sigma_k)^{\mathbb{1}(\phi_i=1)} \mathcal{T}(\mathbf{x}_i|\kappa, \mathbf{M}, V)^{\mathbb{1}(\phi_i=0)}
\end{aligned} \tag{2}$$

Since $p(\phi_i = 1) = 1 - \epsilon$, we can rewrite the last line of the model (2) as the following:

$$p(\mathbf{x}_i|z_i = k, \theta, \Phi, \Theta) = (1-\epsilon)\mathcal{N}(\mathbf{x}_i|\boldsymbol{\mu}_k, \Sigma_k) + \epsilon\mathcal{T}(\mathbf{x}_i|\kappa, \mathbf{M}, V).$$

The total joint probability is

$$
\begin{aligned}
p(\theta,\Theta,X,Z,\Phi) =& p(X,Z,\Phi|\theta,\pi,\epsilon)p(\epsilon|u,v)p(\theta|H)p(\pi|\beta)\\
=&\prod_{i=1}^{n}\prod_{k=1}^{K}\left(\pi_k((1-\epsilon)\mathcal{N}(x_i|\boldsymbol{\mu}_k,\Sigma_k))^{\mathbb{1}(\phi_i=1)}(\epsilon\mathcal{T}(x_i|\kappa,\mathbf{M},V))^{\mathbb{1}(\phi_i=0)}\right)^{\mathbb{1}(z_i=k)}\\
&\cdot\left(\prod_{k=1}^{K}\mathcal{NIW}(H)\right)\cdot Dir(\beta)\cdot\mathcal{B}(u,v).
\end{aligned}
\tag{3}
$$

Before we formally derive an EM algorithm for this model, we derive a few useful quantities. Let $f(\mathbf{x}|\boldsymbol{\mu},\Sigma)$ denote the density of the multivariate normal with mean vector $\boldsymbol{\mu}$ and covariance matrix Σ evaluated at $\mathbf{x}$ and further let $g(\mathbf{x}|\kappa,\mathbf{M},V)$ denote the density of the multivariate T-distribution. We compute that

$$
\begin{aligned}
p(\phi_i=1|z_i=k,\mathbf{x}_i) =&\frac{p(\phi_i=1,\mathbf{x}_i|z_i=k)}{p(\mathbf{x}_i|z_i=k)}\\
=&\frac{p(\mathbf{x}_i|z_i=k,\phi_i=1)P(\phi_i=1|z_i=k)}{p(\mathbf{x}_i|z_i=k)}\\
=&\frac{(1-\epsilon)f(\mathbf{x}_i|\boldsymbol{\mu}_k,\Sigma_k)}{(1-\epsilon)f(\mathbf{x}_i|\boldsymbol{\mu}_k,\Sigma_k)+\epsilon g(\mathbf{x}_i|\kappa,\mathbf{M},V)}.
\end{aligned}
\tag{4}
$$

Likewise we see that,

$$
p(\phi_i=0|z_i=k,\mathbf{x}_i)=\frac{\epsilon f(\mathbf{x}_i|M,V)}{(1-\epsilon)f(\mathbf{x}_i|\boldsymbol{\mu}_k,\Sigma_k)+\epsilon g(\mathbf{x}_i|\kappa,\mathbf{M},V)}.
\tag{5}
$$

Thus

$$
\begin{aligned}
&p(\phi_i=1,z_i=k|\mathbf{x}_i)\\
&=p(\phi_i=1|z_i=k,\mathbf{x}_i)p(z_i=k|\mathbf{x}_i)\\
&=p(\phi_i=1|z_i=k,\mathbf{x}_i)\frac{p(\mathbf{x}_i|z_i=k)p(z_i=k)}{p(\mathbf{x}_i)}\\
&=p(\phi_i=1|z_i=k,\mathbf{x}_i)\frac{\left(p(\mathbf{x}_i|z_i=k,\phi_i=0)p(\phi_i=0)+p(\mathbf{x}_i|z_i=k,\phi_i=1)p(\phi_i=1)\right)p(z_i=k)}{p(\mathbf{x}_i)}
\end{aligned}
\tag{6}
$$

and then substituting values leads to

$$
\begin{aligned}
&\frac{(1-\epsilon)f(\mathbf{x}_i|\boldsymbol{\mu}_k,\Sigma_k)}{(1-\epsilon)f(\mathbf{x}_i|\boldsymbol{\mu}_k,\Sigma_k)+\epsilon g(\mathbf{x}_i|\kappa,\mathbf{M},V)}\frac{\pi_k\left((1-\epsilon)f(\mathbf{x}_i|\boldsymbol{\mu}_k,\Sigma_k)+\epsilon g(\mathbf{x}_i|\kappa,\mathbf{M},V)\right)}{\sum_{k=1}^{K}\pi_k\left((1-\epsilon)f(\mathbf{x}_i|\boldsymbol{\mu}_k,\Sigma_k)+\epsilon g(\mathbf{x}_i|\kappa,\mathbf{M},V)\right)}=\\
&\frac{\pi_k(1-\epsilon)f(\mathbf{x}_i|\boldsymbol{\mu}_k,\Sigma_k)}{\sum_{k=1}^{K}\pi_k\left((1-\epsilon)f(\mathbf{x}_i|\boldsymbol{\mu}_k,\Sigma_k)+\epsilon g(\mathbf{x}_i|\kappa,\mathbf{M},V)\right)}.
\end{aligned}
\tag{7}
$$

We also see that

$$p(\phi_i = 0, z_i = k|\mathbf{x}_i) = \frac{\pi_k \epsilon g(\mathbf{x}_i|\kappa, \mathbf{M}, V)}{\sum_{k=1}^{K} \pi_k \left((1-\epsilon) f(\mathbf{x}_i|\boldsymbol{\mu}_k, \Sigma_k) + \epsilon g(\mathbf{x}_i|\kappa, \mathbf{M}, V)\right)}. \tag{8}$$

We can now formally derive the EM algorithm for this model. First, we compute the expected value of the log-posterior function with respect to the conditional distribution of the latent variable given the observations (under the current estimate of the parameters). For notational convenience we suppress the dependence on the parameters.

$$\begin{aligned}
&Q(\boldsymbol{\theta}|\hat{\boldsymbol{\theta}}) \\
&\quad = E_{Z,\Phi|X,\hat{\boldsymbol{\theta}}}[\log p(\boldsymbol{\theta}; X, Z, \Phi)] \\
&\quad = \sum_{i=1}^{n} E_{Z,\Phi|X,\hat{\boldsymbol{\theta}}}[\log p(\boldsymbol{\theta}; \mathbf{x}_i, z_i, \phi_i)] \\
&\quad = \sum_{i=1}^{n}\sum_{k=1}^{K}\sum_{r=0}^{1} p(z_i = k, \phi_i = r|\mathbf{x}_i) \log(L(\boldsymbol{\theta}_k|\mathbf{x}_i, z_i = k, \phi_i)) + \log(p(\pi) + \sum_{k=1}^{K} \log(p(\boldsymbol{\theta}_k)) \\
&\quad = \sum_{i=1}^{n}\sum_{k=1}^{K}\sum_{r=0}^{1} p(z_i = k, \phi_i = r|\mathbf{x}_i) \log(p(\mathbf{x}_i, z_i = k, \phi_i|\boldsymbol{\theta}_k)) + \log(p(\pi) + \sum_{k=1}^{K} \log(p(\boldsymbol{\theta}_k)) \\
&\quad = Q'(\boldsymbol{\theta}|\hat{\boldsymbol{\theta}}) + D(\boldsymbol{\pi}, \boldsymbol{\theta})
\end{aligned} \tag{9}$$

We note that the equation splits up into a likelihood term Q' plus the log prior D. The coefficient of the first term in the equation above has already been derived and the other term is given by:

$$\begin{aligned}
&p(\mathbf{x}_i, z_i = k, \phi_i)|\boldsymbol{\theta}_k) \\
&\qquad = p(\mathbf{x}_i, \phi_i|\boldsymbol{\theta}_k, z_i = k) p(z_i = k|\boldsymbol{\theta}_k) \\
&\qquad = \pi_k p(\mathbf{x}_i, \phi_i|\boldsymbol{\theta}_k, z_i = k) \\
&\qquad = \pi_k \left(p(\mathbf{x}_i|\boldsymbol{\theta}_k, z_i = k, \phi_i) p(\phi_i|\boldsymbol{\theta}_k, z_i = k)\right) \\
&\qquad = \pi_k \left(((1-\epsilon) f(\mathbf{x}_i|\boldsymbol{\mu}_k, \Sigma_k))^{\phi_i} (\epsilon g(\mathbf{x}_i|\kappa, \mathbf{M}, V))^{1-\phi_i}\right),
\end{aligned} \tag{10}$$

where we used that ϕ_i was a binary random variable. Thus we see that

$$
\begin{aligned}
&Q'(\boldsymbol{\theta}|\hat{\boldsymbol{\theta}}) \\
=&\sum_{i=1}^{n}\sum_{k=1}^{K}\sum_{\Phi} p(z_i=k,\phi_i|\mathbf{x}_i)\log(p(\mathbf{x}_i,z_i=k,\phi_i|\boldsymbol{\theta}_k)) \\
=&\sum_{i=1}^{n}\sum_{k=1}^{K}\sum_{\Phi} p(z_i=k,\phi_i|\mathbf{x}_i)\log(\pi_k((1-\epsilon)f(\mathbf{x}_i|\boldsymbol{\mu}_k,\Sigma_k))^{\phi_i}(\epsilon g(\mathbf{x}_i|\kappa,\mathbf{M},V))^{1-\phi_i}) \\
=&\sum_{i=1}^{n}\sum_{k=1}^{K}\sum_{\Phi} p(z_i=k,\phi_i|\mathbf{x}_i)\left(\log(\pi_k)+\phi_i\log((1-\epsilon)f(\mathbf{x}_i|\boldsymbol{\mu}_k,\Sigma_k))+(1-\phi_i)\log(\epsilon g(\mathbf{x}_i|\kappa,\mathbf{M},V))\right) \\
=&(A)+(B)+(C)+(D)
\end{aligned} \tag{11}
$$

where

$$
\begin{aligned}
(A) &= \sum_{i=1}^{n}\sum_{k=1}^{K} p(z_i=k|\mathbf{x}_i)\log(\pi_k) \\
(B) &= \sum_{i=1}^{n}\sum_{k=1}^{K}\sum_{\Phi} p(z_i=k,\phi_i|\mathbf{x}_i)(\phi_i\log(1-\epsilon)+(1-\phi_i)\log(\epsilon)) \\
(C) &= \sum_{i=1}^{n}\sum_{k=1}^{K}\sum_{\Phi} p(z_i=k,\phi_i|\mathbf{x}_i)\phi_i\log(f(\mathbf{x}_i|\boldsymbol{\mu}_k,\Sigma_k)) \\
(D) &= \sum_{i=1}^{n}\sum_{k=1}^{K}\sum_{\Phi} p(z_i=k,\phi_i|\mathbf{x}_i)(1-\phi_i)\log(g(\mathbf{x}_i|\kappa,\mathbf{M},V)).
\end{aligned} \tag{12}
$$

Then again using that ϕ_i is binary we can make the following simplifications.

$$
\begin{aligned}
(B) &= \sum_{i=1}^{n}\sum_{k=1}^{K} p(z_i=k,\phi_i=1|\mathbf{x}_i)\log(1-\epsilon)+p(z_i=k,\phi_i=0|x_i)\log(\epsilon) \\
(C) &= \sum_{i=1}^{n}\sum_{k=1}^{K} p(z_i=k,\phi_i=1|\mathbf{x}_i)\log(f(\mathbf{x}_i|\boldsymbol{\mu}_k,\Sigma_k)) \\
(D) &= \sum_{i=1}^{n}\sum_{k=1}^{K} p(z_i=k,\phi_i=0|\mathbf{x}_i)\log(g(\mathbf{x}_i|\kappa,\mathbf{M},V)).
\end{aligned} \tag{13}
$$

Terms can now be maximised by considering terms independently because of linearity. Note that the equations **??** and **??** are computed with respect to the current estimated values of the parameters. For convenience set the following notation

$$
\begin{aligned}
a_{ik} =& p(z_i = k, \phi_i = 1|\mathbf{x}_i) \\
b_{ik} =& p(z_i = k, \phi_i = 0|\mathbf{x}_i) \\
w_{ik} =& p(z_i = k|\mathbf{x}_i) = a_{ik} + b_{ik} \\
a_k =& \sum_{i=1}^{n} a_{ik}, a = \sum_{k=1}^{K} a_k \\
b_k =& \sum_{i=1}^{n} b_{ik}, b = \sum_{k=1}^{K} b_k \\
r_k =& \sum_{i=1}^{n} w_{ik}
\end{aligned} \tag{14}
$$

The maximisation step requires finding $argmax_{\boldsymbol{\theta}} Q(\boldsymbol{\theta}|\hat{\boldsymbol{\theta}})$, this can be found for parameter separately for each linear term. To find $\hat{\epsilon}$, we need only consider computing the maximisation step from equation (B). First set $\epsilon_1 = 1 - \epsilon$ and $\epsilon_2 = \epsilon$ and add the log prior term to equation (B). Thus, the required Lagrangian is

$$
\mathcal{L}_\epsilon = a \log(\epsilon_1) + b \log(\epsilon_2) + (u-1)\log(\epsilon_2) + (v-1)\log((\epsilon_1) + \lambda(\epsilon_1 + \epsilon_2 - 1) + constant. \tag{15}
$$

Solving this system leads to

$$
\epsilon = \frac{u + b - 1}{(a+b) + (u+v) - 2}. \tag{16}
$$

To find the MAP estimate for $\boldsymbol{\pi}$, we examine equation (A) and add the log prior. Furthermore we must maximise $\boldsymbol{\pi}$ under the constraint that $\sum_{k=1}^{K} \pi_k = 1$. The Lagrangian for this constrained optimisation problem is the following,

$$
\mathcal{L} = \sum_{i=1}^{n} \sum_{k=1}^{K} w_{ik} \log(\pi_k) - \log(B(\beta)) + \sum_{k=1}^{K} (\beta_k - 1)\log(\pi_k) + \lambda \left(\sum_{k=1}^{K} \pi_k - 1 \right). \tag{17}
$$

The fixed point of this Lagrangian solves the required constrained optimisation problem and $B(\beta)$ denotes the Beta function with parameter β.

$$
\begin{aligned}
\frac{\partial \mathcal{L}}{\partial \pi_k} &= \frac{r_k}{\pi_k} + \frac{\beta_k - 1}{\pi_k} + \lambda = 0 \\
\frac{\partial \mathcal{L}}{\partial \lambda} &= \sum_{k=1}^{K} \pi_k - 1 = 0
\end{aligned} \tag{18}
$$

Solving this pair of equations yields

$$\pi_k = \frac{r_k + \beta_k - 1}{N + \sum \beta_k - K}. \tag{19}$$

To find the posterior mode of the remaining parameters requires some work. First we recall that the normal inverse-Wishart prior is proportional to:

$$\prod_{k=1}^{K} |\Sigma_k|^{\frac{\nu_0+D+2}{2}} \exp\left(-\frac{1}{2}tr(\Sigma_k^{-1}S_0^{-1})\right) \exp\left(-\frac{\lambda_0}{2}tr(\Sigma_k^{-1}(\boldsymbol{\mu}_k - \boldsymbol{\mu}_0)^T(\boldsymbol{\mu}_k - \boldsymbol{\mu}_0))\right). \tag{20}$$

The required equation we are interested in is (C).

$$\begin{aligned}
&\sum_{i=1}^{n}\sum_{k=1}^{K} a_{ik}\log(f(\mathbf{x}_i|\boldsymbol{\mu}_k,\Sigma_k)) \\
&\quad = \sum_{k=1}^{K}\left\{-\sum_{i=1}^{n} a_{ik}\frac{D\log(2\pi)}{2} - \frac{1}{2}\sum_{k=1}^{n} a_{ik}\log|\Sigma_k| - \frac{1}{2}\sum_{i=1}^{n} a_{ik}tr\left(\Sigma_k^{-1}(\mathbf{x}_i - \boldsymbol{\mu}_k)^T(\mathbf{x}_i - \boldsymbol{\mu}_k)\right)\right\} \\
&\quad = \sum_{k=1}^{K}\left\{-a_k\frac{D\log(2\pi)}{2} - \frac{1}{2}a_k\log|\Sigma_k| - \frac{1}{2}tr\left(\Sigma_k^{-1}\sum_{i=1}^{n} a_{ik}(\mathbf{x}_i - \boldsymbol{\mu}_k)^T(\mathbf{x}_i - \boldsymbol{\mu}_k)\right)\right\}.
\end{aligned} \tag{21}$$

Now to derive the M-step objective we remove the constant terms and add on the log prior. This leads to

$$\begin{aligned}
&\sum_{k=1}^{K}\left\{\frac{\nu_0 + D + 2}{2}\log|\Sigma_k| - \frac{1}{2}tr\left(\Sigma_k^{-1}S_0^{-1}\right) - \frac{\lambda_0}{2}tr\left(\Sigma_k^{-1}(\boldsymbol{\mu}_k - \boldsymbol{\mu}_0)^T(\boldsymbol{\mu}_k - \boldsymbol{\mu}_0)\right)\right\} \\
&+ \sum_{k=1}^{K}\left\{-\frac{1}{2}a_k\log|\Sigma_k| - \frac{1}{2}tr\left(\Sigma_k^{-1}\sum_{i=1}^{n} a_{ik}(\mathbf{x}_i - \boldsymbol{\mu}_k)^T(\mathbf{x}_i - \boldsymbol{\mu}_k)\right)\right\}.
\end{aligned} \tag{22}$$

This can be rewritten as

$$\begin{aligned}
&\sum_{k=1}^{K}\left\{\frac{\nu_0 + D + 2 + a_k}{2}\log|\Sigma_k| - \frac{1}{2}tr\left(\Sigma_k^{-1}S_0^{-1}\right) - \frac{\lambda_0}{2}tr\left(\Sigma_k^{-1}(\boldsymbol{\mu}_k - \boldsymbol{\mu}_0)^T(\boldsymbol{\mu}_k - \boldsymbol{\mu}_0)\right)\right\} \\
&+ \sum_{k=1}^{K}\left\{-\frac{1}{2}tr\left(\Sigma_k^{-1}\sum_{i=1}^{n} a_{ik}(\mathbf{x}_i - \boldsymbol{\mu}_k)^T(\mathbf{x}_i - \boldsymbol{\mu}_k)\right)\right\}.
\end{aligned} \tag{23}$$

Now define $\bar{\mathbf{x}}_k = (\sum_{i=i}^{n} a_{ik}\mathbf{x}_i)/a_k$ and note the following algebraic rearrangements.

$$
\begin{aligned}
&\sum_{i=1}^{n} a_{ik}(\mathbf{x}_i - \boldsymbol{\mu}_k)^T(\mathbf{x}_i - \boldsymbol{\mu}_k) \\
&\quad = \sum_{i=1}^{n} a_{ik}\mathbf{x}_i^T\mathbf{x}_i - \boldsymbol{\mu}_k^T\mathbf{x}_i - \mathbf{x}_i^T\boldsymbol{\mu}_k + \boldsymbol{\mu}_k^T\boldsymbol{\mu}_k \\
&\quad = \sum_{i=1}^{n} a_{ik}\mathbf{x}_i^T\mathbf{x}_i - \boldsymbol{\mu}_k^T\sum_{i=1}^{n} a_{ik}\mathbf{x}_i - \left(\sum_{i=1}^{n} a_{ik}\mathbf{x}_i^T\right)\boldsymbol{\mu}_k + a_k\boldsymbol{\mu}_k^T\boldsymbol{\mu}_k \\
&\quad = \sum_{i=1}^{n} a_{ik}\mathbf{x}_i^T\mathbf{x}_i - a_k\boldsymbol{\mu}_k^T\bar{\mathbf{x}}_k - a_k\bar{\mathbf{x}}_k^T\boldsymbol{\mu}_k + a_k\boldsymbol{\mu}_k^T\boldsymbol{\mu}_k \\
&\quad = \sum_{i=1}^{n} a_{ik}\mathbf{x}_i^T\mathbf{x}_i - a_k\bar{\mathbf{x}}_k^T\bar{\mathbf{x}}_k + a_k(\bar{\mathbf{x}}_k - \boldsymbol{\mu}_k)^T(\bar{\mathbf{x}}_k - \boldsymbol{\mu}_k) \\
&\quad = \sum_{i=1}^{n} a_{ik}(\mathbf{x}_i - \bar{\mathbf{x}}_k)^T(\mathbf{x}_i - \bar{\mathbf{x}}_k) + a_k(\bar{\mathbf{x}}_k - \boldsymbol{\mu}_k)^T(\bar{\mathbf{x}}_k - \boldsymbol{\mu}_k)
\end{aligned} \tag{24}
$$

This allows us to rewrite equation 23 as

$$
\begin{aligned}
&\sum_{k=1}^{K}\left\{\frac{\nu_0 + D + 2 + a_k}{2}\log|\Sigma_k| - \frac{1}{2}tr\left(\Sigma_k^{-1}\left(S_0^{-1} + \sum_{i=1}^{n} a_{ik}(\mathbf{x}_i - \bar{\mathbf{x}}_k)^T(\mathbf{x}_i - \bar{\mathbf{x}}_k)\right)\right)\right\} \\
&+\sum_{k=1}^{K}\left\{-\frac{1}{2}tr\left(\Sigma_k^{-1}\left(\lambda_0(\boldsymbol{\mu}_k - \boldsymbol{\mu}_0)^T(\boldsymbol{\mu}_k - \boldsymbol{\mu}_0)\right) + a_k(\bar{\mathbf{x}}_k - \boldsymbol{\mu}_k)^T(\bar{\mathbf{x}}_k - \boldsymbol{\mu}_k)\right)\right\}
\end{aligned} \tag{25}
$$

This can be written as:

$$
\sum_{k=1}^{K}\left\{\frac{\nu_k + D + 2}{2}\log|\Sigma_k| - \frac{1}{2}tr\left(\Sigma_k^{-1}S_k^{-1}\right) - \frac{1}{2}tr\left(\Sigma_k^{-1}\left(\lambda_k(\boldsymbol{\mu}_k - \boldsymbol{m}_k)^T(\boldsymbol{\mu}_k - \boldsymbol{m}_k)\right)\right)\right\} \tag{26}
$$

where,

$$
\begin{aligned}
\lambda_k &= \lambda_0 + a_k \\
\nu_k &= \nu_0 + a_k \\
\boldsymbol{m}_k &= \frac{a_k\bar{\mathbf{x}}_k + \lambda_0\boldsymbol{\mu}_0}{\lambda_k} \\
S_k^{-1} &= S_0^{-1} + \frac{\lambda_0 a_k}{\lambda_k}(\bar{\mathbf{x}}_k - \boldsymbol{\mu}_0)^T(\bar{\mathbf{x}}_k - \boldsymbol{\mu}_0) + \sum_{i=1}^{n} a_{ik}(\mathbf{x}_i - \bar{\mathbf{x}}_k)^T(\mathbf{x}_i - \bar{\mathbf{x}}_k)
\end{aligned} \tag{27}
$$

Thus the parameters of the posterior mode are:

$$
\begin{aligned}
\hat{\boldsymbol{\mu}}_k =& \boldsymbol{m}_k \\
\hat{\Sigma}_k =& \frac{1}{\nu_k + D + 2} S_k^{-1}
\end{aligned}
\tag{28}
$$

To summarise the EM algorithm, we iterate between the two steps:

E-Step: Given the current parameters compute the values given by equations (14), with formulas provided in equations (**??**) and (**??**).

M-Step: Compute

$$
\epsilon = \frac{u + b - 1}{(a + b) + (u + v) - 2},
$$

and

$$
\pi_k = \frac{r_k + \beta_k - 1}{N + \sum \beta_k - K},
$$

as well as

$$
\bar{\mathbf{x}}_k = \frac{1}{a_k} \left(\sum_{i=i}^{n} a_{ik} \mathbf{x}_i \right)
$$

Compute the MAP estimates given by equations (28). These estimates are then used in the following iteration of the E-step. Iterate until $|Q(\boldsymbol{\theta}|\boldsymbol{\theta}_t) - Q(\boldsymbol{\theta}|\boldsymbol{\theta_{t-1}})| < \delta$ for some pre-specified $\delta > 0$.

S2 Derivation of collapsed Gibbs sampler for TAGM model

To derive the Gibbs sampler we write down all the conditional probabilities. Then, exploiting conjugacy, we can marginalise parameters in the model. Recall the total joint probability is the following:

$$
\begin{aligned}
p(\boldsymbol{\theta}, \Theta, X, Z, \Phi) =& p(X, Z, \Phi|\theta, \boldsymbol{\pi}, \epsilon) p(\epsilon|u, v) p(\theta|H) p(\boldsymbol{\pi}|\beta) \\
=& \prod_{i=1}^{n} \prod_{k=1}^{K} \left(\pi_k ((1-\epsilon) \mathcal{N}(\mathbf{x}_i|\boldsymbol{\mu}_k, \Sigma_k))^{\mathbb{1}(\phi_i=1)} (\epsilon \mathcal{T}(\mathbf{x}_i|\kappa, \mathbf{M}, V))^{\mathbb{1}(\phi_i=0)} \right)^{\mathbb{1}(z_i=k)} \\
& \cdot \left(\prod_{k=1}^{K} \mathcal{NIW}(H) \right) \cdot Dir(\beta) \cdot \mathcal{B}(u, v).
\end{aligned}
\tag{29}
$$

Suppose we know the hidden latent component allocations z_i and outlier allocations ϕ_i. Then we could sample from the a required normal distribution. The conditional probability of the parameters given the allocations is given by:

$$
p(\theta_k|X, Z, \Phi, \theta_{-k}, \beta, u, v, H) \propto p_0(\theta_k) \prod_{i=1}^{n} N(\mathbf{x}_i|\boldsymbol{\mu}_k, \Sigma_k)^{\mathbb{1}(\phi_i=1)}. \tag{30}
$$

The prior is conjugate and so the posterior belongs to the same parametric family as the prior, a NIW distribution, and so the parameters can be updated as follows:

$$\begin{aligned} m_k &= \frac{n_k \bar{\mathbf{x}}_k + \lambda_0 \boldsymbol{\mu}_0}{\lambda_k} \\ \lambda_k &= \lambda_0 + n_k \\ \nu_k &= \nu_0 + n_k \\ S_k &= S_0 + \sum_{i: z_i = k, \phi_i = 1} (\mathbf{x}_i - \bar{\mathbf{x}})^T (\mathbf{x}_i - \bar{\mathbf{x}}) + \frac{\lambda_0 n_k}{\lambda_k} (\bar{\mathbf{x}} - \boldsymbol{\mu}_0)^T (\bar{\mathbf{x}} - \boldsymbol{\mu}_0), \end{aligned} \tag{31}$$

where $n_k = |\{\mathbf{x}_i | z_i = k, \phi_i = 1\}|$. Now we write down the conditional of the component allocations

$$p(z_i = k | X, z_{-i}, \Phi, \theta, \beta, u, v, H) \propto p_0(z_i = k | z_{-i}, \beta) p(\mathbf{x}_i | \mathbf{x}_{-i}, z_{-i}, z_i = k, \Phi, H). \tag{32}$$

The first term in this equation is

$$p_0(z_i = k | z_{-i}, \beta) = \frac{p(z_i = k, z_{-i} | \beta)}{p(z_{-i} | \beta)} = \frac{p(Z | \beta)}{p(z_{-i} | \beta)}. \tag{33}$$

To calculate the numerator we proceed by marginalising over $\boldsymbol{\pi}$ as follows

$$p(Z | \beta) = \int p(z | \boldsymbol{\pi}) p(\boldsymbol{\pi} | \beta) d\boldsymbol{\pi} = \frac{\Gamma(\beta)}{\Gamma(n + \beta)} \prod_{k=1}^{K} \frac{\Gamma(n_k + \beta_k)}{\Gamma(\beta_k)}. \tag{34}$$

Hence, we arrive at the following probability:

$$p_0(z_i = k | z_{-i}, \beta) = \frac{n_{k \setminus i} + \beta_k}{n + \sum \beta_k - 1}. \tag{35}$$

The conditional for the second term of 32 is more tricky. First note the following conditional distributions

$$\begin{aligned} &\mathbf{x}_i | z_i = k, X_{k \setminus i}, \phi_i = 1, \Phi, z_{-i} \sim \mathcal{N}(\mathbf{x}_i | \theta_k) \\ &\mathbf{x}_i | z_i = k, X_{k \setminus i}, \phi_i = 0, \Phi, z_{-i} \sim \mathcal{T}(\mathbf{x}_i | \kappa, \mathbf{M}, V), \\ &\mathbf{x}_i | z_i = k, X_{k \setminus i}, \phi_i, \Phi, z_{-i} \sim N(\mathbf{x}_i | \theta_k)^{\mathbb{1}(\phi_i = 1)} \mathcal{T}(\mathbf{x}_i |, \kappa, \mathbf{M}, V)^{\mathbb{1}(\phi_i = 0)}, \end{aligned} \tag{36}$$

where we denote $X_{k \setminus i}$ as the observations associated with class k, besides x_i. Now, we first note that:

$$p(\mathbf{x}_i | z_i = k, X_{k \setminus i}, \phi_i, \Phi, H, z_{-i}) = p(\mathbf{x}_i | X_{k \setminus i}, \phi_i, \Phi, H) = \frac{p(\mathbf{x}_i, X_{k \setminus i} | \phi_i, \Phi, H)}{p(X_{k \setminus i} | \phi_i, \Phi, H)}. \tag{37}$$

Thus, we find an equation for the numerator, using the fact that terms associated with $\phi_i = 0$ do not depend on k and thus can be absorbed into the normalising constant.

$$p(X_k|\phi_i, \Phi, H) \propto \prod_{i:\phi_i=1} \int p(\mathbf{x}_i|z_i = k, \Phi, H, \theta_k)p(\theta_k|H)d\theta_k. \tag{38}$$

This is the marginal likelihood of the data. Thus the ratio in 37 is the posterior predictive which is given by the non-centred T-distribution with formula given by:

$$\mathcal{T}\left(v_k - d + 1, m_k, \frac{(1+\lambda_k)S_k}{\lambda_k(v_k - d + 1)}\right).$$

Thus, we can compute the following:

$$\begin{aligned} p(z_i = k|X, z_{-i}, \Phi, \theta, \beta, u, v, H) &\propto p_0(z_i = k|z_{-i}, \beta)p(\mathbf{x}_i|\mathbf{x}_{-i}, z_{-i}, \Phi, z_i = k, H) \\ &= \frac{n_{k\setminus i} + \beta_k}{n + \sum \beta_k - 1}\mathcal{T}\left(\mathbf{x}_i|v_k - d + 1, m_k, \frac{(1+\lambda_k)S_k}{\lambda_k(v_k - d + 1)}\right). \end{aligned} \tag{39}$$

It remains to compute the conditional for the ϕ_i. By first recalling that ϕ_i is binary we see that

$$p(\phi_i|X, Z, \theta, \beta, u, v, H) \propto p_0(\phi_i) \prod_{i=1}^{n} N(\mathbf{x}_i|\theta_{z_i})^{\mathbb{1}(\phi_i=1)} T(\mathbf{x}_i|\kappa, M, V)^{\mathbb{1}(\phi_i=0)} \tag{40}$$

can be written as

$$\begin{aligned} p(\phi_i = 1|X, Z, \theta, \phi_{-i}, \beta, u, v, H) \propto p_0(\phi_i = 1|\phi_{-i}, u, v)p(\mathbf{x}_i|\mathbf{x}_{-i}, \phi_i = 1, Z, \theta, \Phi, \beta, u, v, H), \\ p(\phi_i = 0|X, Z, \theta, \phi_{-i}, \beta, u, v, H) \propto p_0(\phi_i = 0|\phi_{-i}, u, v)p(\mathbf{x}_i|\mathbf{x}_{-i}, \phi_i = 0, Z, \theta, \Phi, \beta, u, v, H). \end{aligned} \tag{41}$$

First we need to compute a formula for $p_0(\phi_i|\phi_{-i}, u, v)$. First we see that

$$p_0(\phi_i|\phi_{-i}, u, v) = \frac{p(\Phi|u, v)}{p(\phi_{-i}|u, v)}. \tag{42}$$

The numerator can be computed by marginalising over ϵ:

$$p(\Phi|u, v) = \int p(\Phi|\epsilon)p(\epsilon|u, v)d\epsilon. \tag{43}$$

We denote $\sum \mathbb{1}(\phi_i = 1) = \tau_1$ and $\sum \mathbb{1}(\phi_i = 1) = \tau_0 = 1 - \tau_1$. Then it is easy to see that

$$\begin{aligned} p(\Phi|u, v) &= \int p(\Phi|\epsilon)p(\epsilon|u, v)d\epsilon \\ &= \frac{1}{B(u, v)} \int (1-\epsilon)^{\tau_1 + v - 1}\epsilon^{\tau_0 + u - 1}d\epsilon \\ &= \frac{B(\tau_0 + u, \tau_1 + v)}{B(u, v)}. \end{aligned} \tag{44}$$

Hence,

$$p(\phi_i = 1|\phi_{-i}, u, v) = \frac{B(\tau_0 + u, \tau_1 + v)}{B(u,v)} \cdot \frac{B(u,v)}{B(\tau_0 + u, \tau_1 + v - 1)} \\ = \frac{\tau_1 + v - 1}{n + u + v - 1}, \tag{45}$$

where $n = \tau_1 + \tau_2$. In general,

$$p(\phi_i = s|\phi_{-i}, u, v) = \frac{\tau_{s\setminus i} + v^s u^{1-s}}{n + u + v - 1}. \tag{46}$$

Now we return to computing $p(\mathbf{x}_i|\mathbf{x}_{-i}, Z, \theta, \phi_i = 1, \Phi, \beta, u, v, H)$. First we see that

$$p(\mathbf{x}_i|\mathbf{x}_{-i}, Z, \theta, \phi_i = 1, \Phi, \beta, u, v, H) = \frac{p(X|Z, \theta, \phi_i = 1, \Phi, \beta, u, v, H)}{p(\mathbf{x}_{-i}|Z, \theta, \phi_i = 1, \Phi, \beta, u, v, H)}. \tag{47}$$

Thus if we integrate over the parameters, we would have a ratio of marginal likelihoods giving the posterior predictive which is a non-centred T-distribution:

$$p(\mathbf{x}_i|\mathbf{x}_{-i}, Z, \theta, \phi_i = 1, \Phi, \beta, u, v, H) = \mathcal{T}\left(v_k - d + 1, m_k, \frac{(1+\lambda_k)S_k}{\lambda_k(v_k - d + 1)}\right). \tag{48}$$

In the other case that $\phi = 0$, we have that

$$p(x_i|x_{-i}, Z, \theta, \phi_i = 0, \Phi, \beta, u, v, H) = \mathcal{T}(x_i|\kappa, \mathbf{M}, V). \tag{49}$$

Thus we can compute:

$$p(\phi_i|X, Z, \theta, \phi_{-i}, \beta, u, v, H) \tag{50}$$

and sample from the required distribution. Thus, we can summarise the collapsed Gibbs sampler as follows:

1. Update the priors with the labelled data
2. For the unlabelled observations, in turn, compute the probability of assigning to each component
3. Sample a label according to this probability
4. Compute the probability of belonging to this class or the outlier component
5. Sample an indicator to a class specific component or the outlier component
6. If we assign to the class specific component update the class specific posterior distribution with the statistics of this observation
7. Update other posteriors as appropriate.

8. Once all unlabelled observations have a been assigned, consider the observations sequentially, removing the statistics from the posteriors and then performing steps 2-7. We repeat this process for all unlabelled observations.

9. repeat 7-8 until convergence of the Markov-chain.

The computational bottleneck in the algorithm is computing the posterior updates for the parameters

$$
\begin{aligned}
m_k =& \frac{n_k \bar{\mathbf{x}}_k + \lambda_0 \boldsymbol{\mu}_0}{\lambda_k} \\
\lambda_k =& \lambda_0 + n_k \\
\nu_k =& \nu_0 + n_k \\
S_k =& S_0 + \sum_{i:z_i=k,\phi_i=1} (\mathbf{x}_i - \bar{\mathbf{x}})^T (\mathbf{x}_i - \bar{\mathbf{x}}) + \frac{\lambda_0 n_k}{\lambda_k} (\bar{\mathbf{x}} - \boldsymbol{\mu}_0)^T (\bar{\mathbf{x}} - \boldsymbol{\mu}_0),
\end{aligned}
\tag{51}
$$

We first note that

$$
S_k = S_0 + \sum_{i:z_i=k,\phi_i=1} \mathbf{x}_i^T \mathbf{x}_i + \lambda_0 \boldsymbol{\mu}_0^T \boldsymbol{\mu}_0 - \lambda_k \boldsymbol{\mu}_k^T \boldsymbol{\mu}_k \tag{52}
$$

Let us denote $T = \sum_{i:z_i=k,\phi_i=1} \mathbf{x}_i^T \mathbf{x}_i$. Thus we can derive a set of iterative updates to speed up computation when adding/removing statistics from clusters. More precisely, indicating updated posterior parameters by a prime, if we remove statistics of observation i from cluster k, we see that

$$
\begin{aligned}
m'_k =& \frac{\lambda_k m_k - \mathbf{x}_i}{\lambda_k - 1} \\
\lambda'_k =& \lambda_k - 1 \\
\nu'_k =& \nu_k - 1 \\
T' =& T - \mathbf{x}_i^T \mathbf{x}_i \\
S'_k =& S_0 + T' + \lambda_0 \boldsymbol{\mu}_0^T \boldsymbol{\mu}_0 - \lambda_k m_k'^T m'_k.
\end{aligned}
\tag{53}
$$

Likewise if we add the statistics of observation i to cluster k, we see that

$$
\begin{aligned}
m'_k =& \frac{\lambda_k m_k + \mathbf{x}_i}{\lambda_k + 1} \\
\lambda'_k =& \lambda_k + 1 \\
\nu'_k =& \nu_k + 1 \\
T' =& T + \mathbf{x}_i^T \mathbf{x}_i \\
S'_k =& S_0 + T' + \lambda_0 \boldsymbol{\mu}_0^T \boldsymbol{\mu}_0 - \lambda_k m_k'^T m'_k.
\end{aligned}
\tag{54}
$$

S3 Convergence diagnostics of EM algorithm

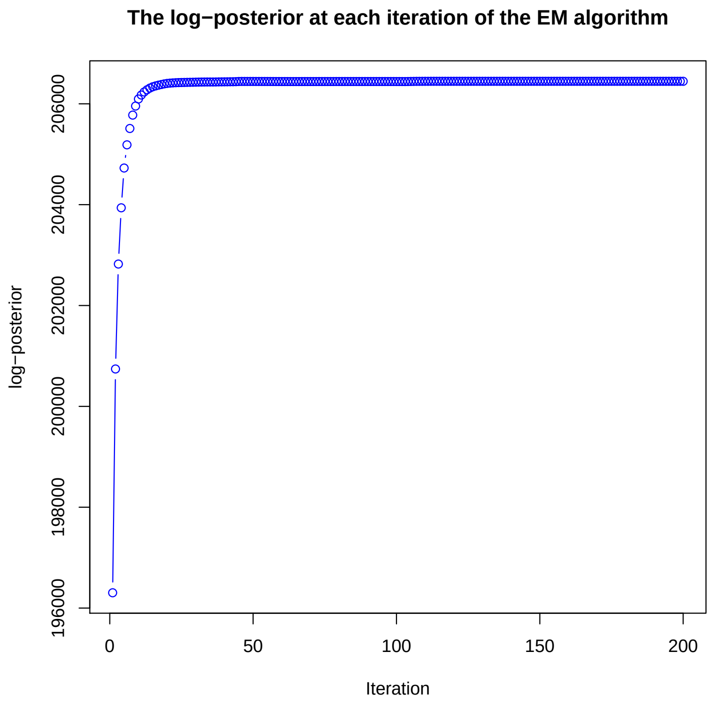

Figure A: Plot of the log-posterior at each iteration of the EM algorithm to demonstrate monotonicity and convergence

S4 Trace plots for assessing MCMC convergence

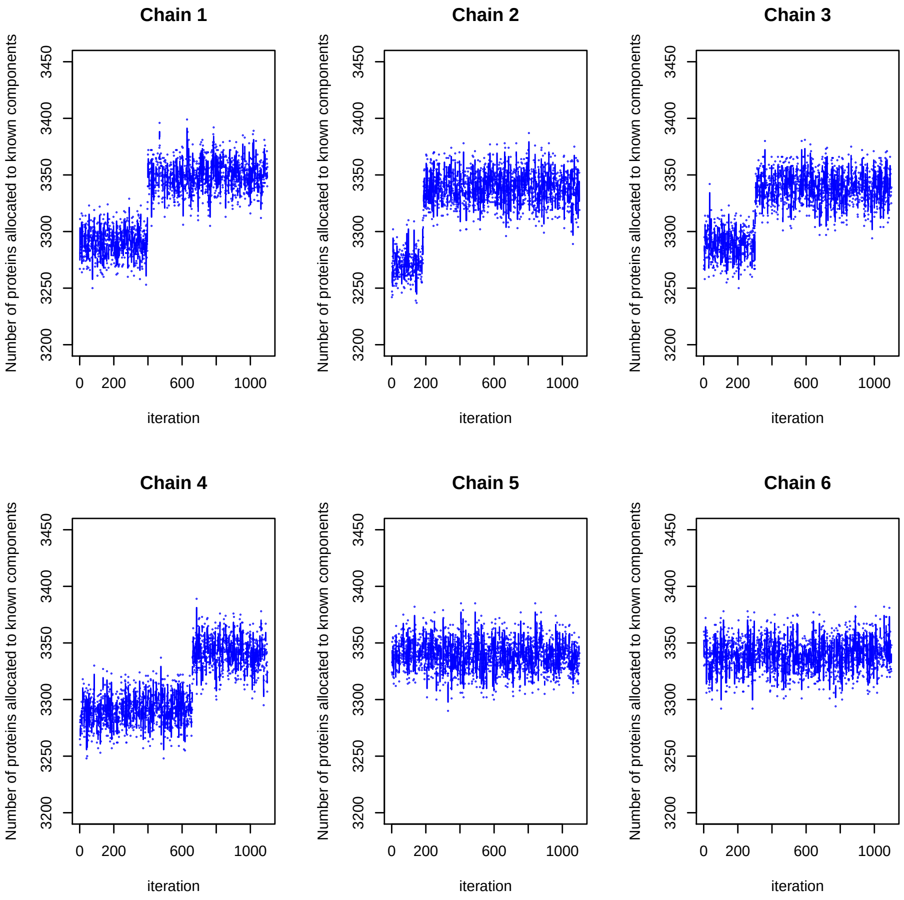

Figure B: Trace plots of the number of proteins allocated to the known components in each of 6 parallel MCMC runs. Chain 4 is discarded because of lack of convergence. 600 samples are retained from remaining chains and pooled.

S5 F1 t-tests

	SVM	KNN	MAP
KNN	2.7E-03		
MAP	3.3E-02	3.4E-01	
MCMC	3.4E-01	3.3E-02	2.3E-01

Table A1: Adjusted P-values for pairwise T-tests for Macro F-1 score classifier evaluation on the Drosophila dataset

	SVM	KNN	MAP
KNN	1.2E-02		
MAP	2.7E-01	1.5E-01	
MCMC	4.9E-01	1.9E-03	1.1E-01

Table A2: Adjusted P-values for pairwise T-tests for Macro F-1 score classifier evaluation on the Chicken DT40 dataset

	SVM	KNN	MAP
KNN	1.0E+00		
MAP	1.0E+00	1.0E+00	
MCMC	3.3E-01	6.0E-02	1.1E-05

Table A3: Adjusted P-values for pairwise T-tests for Macro F-1 score classifier evaluation on the mouse dataset

	SVM	KNN	MAP
KNN	1.4E-35		
MAP	3.3E-06	6.7E-21	
MCMC	8.0E-59	3.2E-91	2.4E-70

Table A4: Adjusted P-values for pairwise T-tests for Macro F-1 score classifier evaluation on the HeLa dataset

	SVM	KNN	MAP
KNN	1.3E-02		
MAP	4.3E-04	3.3E-09	
MCMC	5.8E-01	3.5E-03	3.1E-03

Table A5: Adjusted P-values for pairwise T-tests for Macro F-1 score classifier evaluation on the U2-OS dataset

	SVM	KNN	MAP
KNN	2.2E-08		
MAP	1.0E-34	6.8E-14	
MCMC	7.4E-05	5.3E-02	1.0E-20

Table A6: Adjusted P-values for pairwise T-tests for Macro F-1 score classifier evaluation on the HeLa wild (Hirst et al.) dataset

	SVM	KNN	MAP
KNN	5.3E-02		
MAP	1.7E-23	7.9E-27	
MCMC	9.1E-02	5.8E-04	1.8E-19

Table A7: Adjusted P-values for pairwise T-tests for Macro F-1 score classifier evaluation on the HeLa KO1 (Hirst et al.) dataset

	SVM	KNN	MAP
KNN	1.3E-01		
MAP	1.1E-55	1.1E-55	
MCMC	1.0E-18	6.3E-22	2.0E-26

Table A8: Adjusted P-values for pairwise T-tests for Macro F-1 score classifier evaluation on the HeLa KO2 (Hirst et al.) dataset

	SVM	KNN	MAP
KNN	9.6E-02		
MAP	4.1E-07	1.1E-09	
MCMC	2.8E-27	1.0E-28	6.3E-10

Table A9: Adjusted P-values for pairwise T-tests for Macro F-1 score classifier evaluation on the Primary Fibroblasts Mock 24hpi dataset

	SVM	KNN	MAP
KNN	6.6E-07		
MAP	1.3E-10	2.0E-01	
MCMC	1.6E-05	2.0E-01	6.2E-03

Table A10: Adjusted P-values for pairwise T-tests for Macro F-1 score classifier evaluation on the Primary Fibroblasts Mock 48hpi dataset

	SVM	KNN	MAP
KNN	3.9E-03		
MAP	9.5E-01	8.6E-03	
MCMC	6.4E-02	3.0E-01	8.6E-02

Table A11: Adjusted P-values for pairwise T-tests for Macro F-1 score classifier evaluation on the Primary Fibroblasts Mock 72hpi dataset

	SVM	KNN	MAP
KNN	8.6E-03		
MAP	1.1E-02	8.6E-01	
MCMC	3.7E-06	1.6E-02	3.3E-02

Table A12: Adjusted P-values for pairwise T-tests for Macro F-1 score classifier evaluation on the Primary Fibroblasts Mock 96hpi dataset

	SVM	KNN	MAP
KNN	1.9E-23		
MAP	1.4E-02	2.3E-34	
MCMC	3.8E-07	1.6E-81	2.0E-02

Table A13: Adjusted P-values for pairwise T-tests for Macro F-1 score classifier evaluation on the Primary Fibroblasts Mock 120hpi dataset

	SVM	KNN	MAP
KNN	4.6E-01		
MAP	2.6E-05	1.7E-04	
MCMC	1.7E-04	1.3E-03	5.5E-01

Table A14: Adjusted P-values for pairwise T-tests for Macro F-1 score classifier evaluation on the Primary Fibroblasts HCMV 24hpi dataset

	SVM	KNN	MAP
KNN	1.0E-02		
MAP	4.6E-01	1.5E-03	
MCMC	1.2E-02	7.3E-01	1.5E-03

Table A15: Adjusted P-values for pairwise T-tests for Macro F-1 score classifier evaluation on the Primary Fibroblasts HCMV 48hpi dataset

	SVM	KNN	MAP
KNN	5.5E-02		
MAP	9.5E-06	3.4E-02	
MCMC	1.1E-01	6.2E-01	6.4E-03

Table A16: Adjusted P-values for pairwise T-tests for Macro F-1 score classifier evaluation on the Primary Fibroblasts HCMV 72hpi dataset

	SVM	KNN	MAP
KNN	2.8E-01		
MAP	2.6E-09	7.2E-08	
MCMC	4.2E-10	5.6E-09	5.7E-01

Table A17: Adjusted P-values for pairwise T-tests for Macro F-1 score classifier evaluation on the Primary Fibroblasts HCMV 96hpi dataset

	SVM	KNN	MAP
KNN	2.3E-04		
MAP	7.1E-04	3.8E-10	
MCMC	1.4E-01	5.7E-02	6.0E-05

Table A18: Adjusted P-values for pairwise T-tests for Macro F-1 score classifier evaluation on the Primary Fibroblasts HCMV 120hpi dataset

	SVM	KNN	MAP
KNN	6.7E-06		
MAP	6.3E-05	4.4E-01	
MCMC	4.4E-01	6.7E-06	8.3E-05

Table A19: Adjusted P-values for pairwise T-tests for Macro F-1 score classifier evaluation on the E14TG2a dataset

S6 Quadratic loss t-tests

	SVM	KNN	MAP
KNN	5.9E-13		
MAP	1.1E-04	9.6E-124	
MCMC	2.2E-23	3.3E-58	5.9E-171

Table B1: Adjusted P-values for pairwise T-tests for Quadratic Loss classifier evaluation on the Drosphila dataset

	SVM	KNN	MAP
KNN	3.2E-08		
MAP	1.7E-26	1.3E-128	
MCMC	4.2E-13	8.8E-37	7.0E-135

Table B2: Adjusted P-values for pairwise T-tests for Quadratic Loss classifier evaluation on the Chicken DT40 dataset

	SVM	KNN	MAP
KNN	5.5E-14		
MAP	3.0E-25	6.3E-128	
MCMC	7.4E-26	1.7E-129	1.6E-14

Table B3: Adjusted P-values for pairwise T-tests for Quadratic Loss classifier evaluation on the mouse dataset

	SVM	KNN	MAP
KNN	1.2E-02		
MAP	9.4E-07	7.4E-86	
MCMC	5.5E-08	2.7E-89	2.4E-12

Table B4: Adjusted P-values for pairwise T-tests for Quadratic Loss classifier evaluation on the HeLa dataset

	SVM	KNN	MAP
KNN	6.8E-02		
MAP	7.4E-17	1.1E-73	
MCMC	1.4E-20	6.7E-81	8.3E-41

Table B5: Adjusted P-values for pairwise T-tests for Quadratic Loss classifier evaluation on the U2-OS dataset

	SVM	KNN	MAP
KNN	2.3E-92		
MAP	9.0E-13	2.4E-83	
MCMC	6.6E-19	3.0E-81	1.1E-01

Table B6: Adjusted P-values for pairwise T-tests for Quadratic Loss classifier evaluation on the HeLa wild (Hirst et al.) dataset

	SVM	KNN	MAP
KNN	5.2E-97		
MAP	1.4E-02	1.2E-90	
MCMC	2.3E-09	7.0E-95	2.2E-02

Table B7: Adjusted P-values for pairwise T-tests for Quadratic Loss classifier evaluation on the HeLa KO1 (Hirst et al.) dataset

	SVM	KNN	MAP
KNN	8.9E-93		
MAP	3.1E-01	8.1E-91	
MCMC	9.0E-06	1.5E-83	8.9E-05

Table B8: Adjusted P-values for pairwise T-tests for Quadratic Loss classifier evaluation on the HeLa KO2 (Hirst et al.) dataset

	SVM	KNN	MAP
KNN	6.1E-13		
MAP	1.4E-18	4.4E-81	
MCMC	3.2E-18	7.2E-77	5.9E-03

Table B9: Adjusted P-values for pairwise T-tests for Quadratic Loss classifier evaluation on the Primary Fibroblasts Mock 24hpi dataset

	SVM	KNN	MAP
KNN	6.1E-18		
MAP	3.6E-24	2.2E-57	
MCMC	1.4E-24	3.6E-61	3.6E-04

Table B10: Adjusted P-values for pairwise T-tests for Quadratic Loss classifier evaluation on the Primary Fibroblasts Mock 48hpi dataset

	SVM	KNN	MAP
KNN	1.2E-15		
MAP	4.5E-23	2.5E-89	
MCMC	4.2E-23	5.1E-91	4.4E-01

Table B11: Adjusted P-values for pairwise T-tests for Quadratic Loss classifier evaluation on the Primary Fibroblasts Mock 72hpi dataset

	SVM	KNN	MAP
KNN	1.8E-13		
MAP	1.4E-20	3.6E-126	
MCMC	5.0E-20	1.5E-109	5.3E-07

Table B12: Adjusted P-values for pairwise T-tests for Quadratic Loss classifier evaluation on the Primary Fibroblasts Mock 96hpi dataset

	SVM	KNN	MAP
KNN	6.7E-14		
MAP	1.0E-19	2.6E-45	
MCMC	8.0E-20	2.4E-45	2.5E-02

Table B13: Adjusted P-values for pairwise T-tests for Quadratic Loss classifier evaluation on the Primary Fibroblasts Mock 120hpi dataset

	SVM	KNN	MAP
KNN	6.0E-22		
MAP	2.8E-27	6.4E-53	
MCMC	1.4E-27	1.5E-56	3.0E-03

Table B14: Adjusted P-values for pairwise T-tests for Quadratic Loss classifier evaluation on the Primary Fibroblasts HCMV 24hpi dataset

	SVM	KNN	MAP
KNN	1.9E-26		
MAP	1.3E-33	2.7E-84	
MCMC	1.3E-33	2.7E-84	6.0E-01

Table B15: Adjusted P-values for pairwise T-tests for Quadratic Loss classifier evaluation on the Primary Fibroblasts HCMV 48hpi dataset

	SVM	KNN	MAP
KNN	6.3E-20		
MAP	1.9E-25	2.7E-57	
MCMC	1.2E-25	3.4E-58	1.5E-02

Table B16: Adjusted P-values for pairwise T-tests for Quadratic Loss classifier evaluation on the Primary Fibroblasts HCMV 72hpi dataset

	SVM	KNN	MAP
KNN	1.7E-25		
MAP	9.3E-32	1.9E-56	
MCMC	9.3E-32	1.2E-54	7.1E-01

Table B17: Adjusted P-values for pairwise T-tests for Quadratic Loss classifier evaluation on the Primary Fibroblasts HCMV 96hpi dataset

	SVM	KNN	MAP
KNN	6.5E-25		
MAP	5.3E-32	1.1E-71	
MCMC	7.1E-32	8.4E-71	5.7E-02

Table B18: Adjusted P-values for pairwise T-tests for Quadratic Loss classifier evaluation on the Primary Fibroblasts HCMV 120hpi dataset

	SVM	KNN	MAP
KNN	4.7E-04		
MAP	4.7E-21	1.5E-103	
MCMC	3.3E-12	1.8E-57	1.3E-137

Table B19: Adjusted P-values for pairwise T-tests for Quadratic Loss classifier evaluation on the E14TG2a dataset

S7 GO enrichment analysis figures

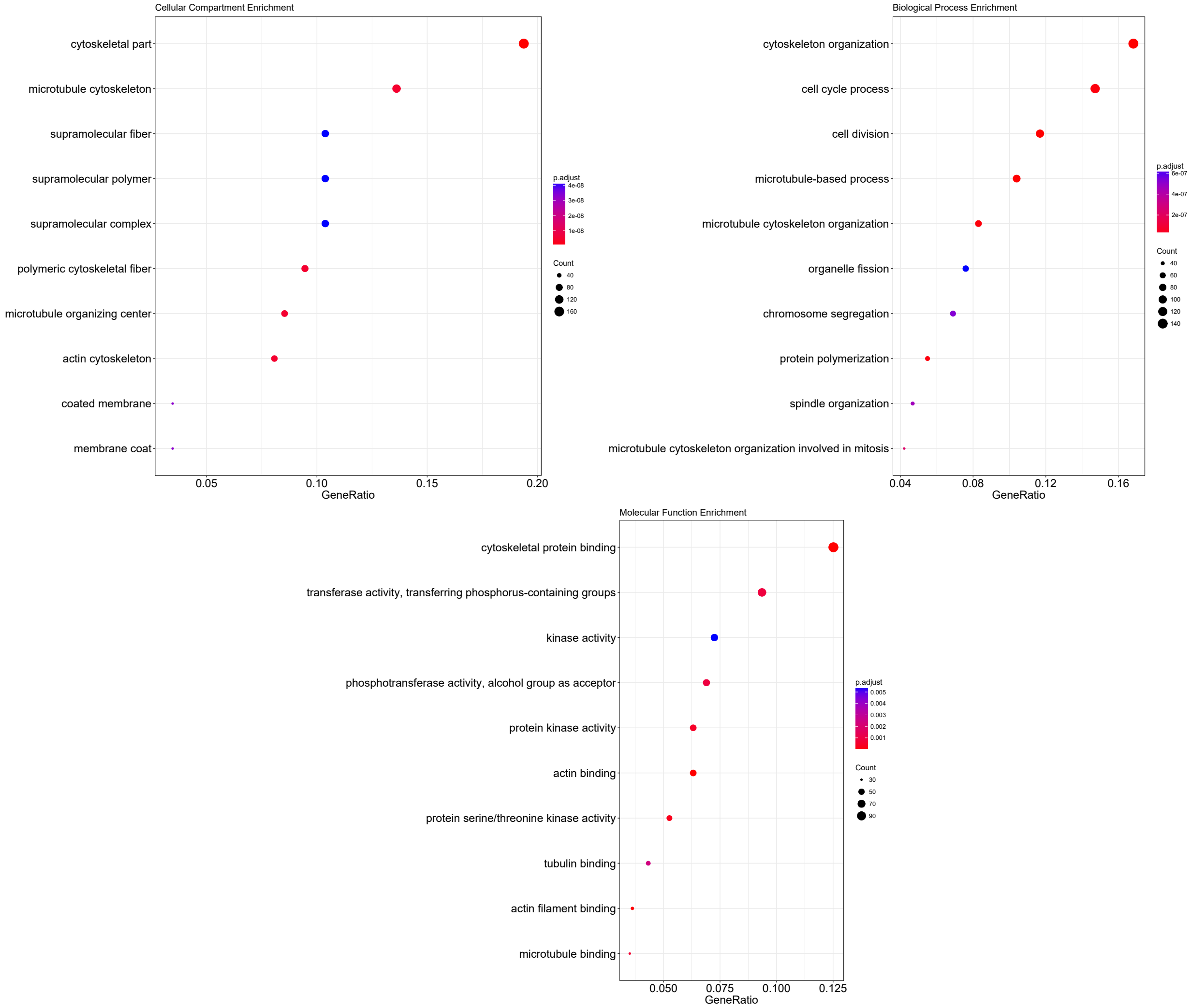

Figure C: Gene Ontology over representation analysis on outlier proteins - that is proteins allocated with less than probability 0.95. We analyse the enrichment of terms in the cellular compartment, biological process, and molecular function ontologies. We display the top 10 significant results in the dotplots.

S8 Comparison of MCMC and MAP allocations

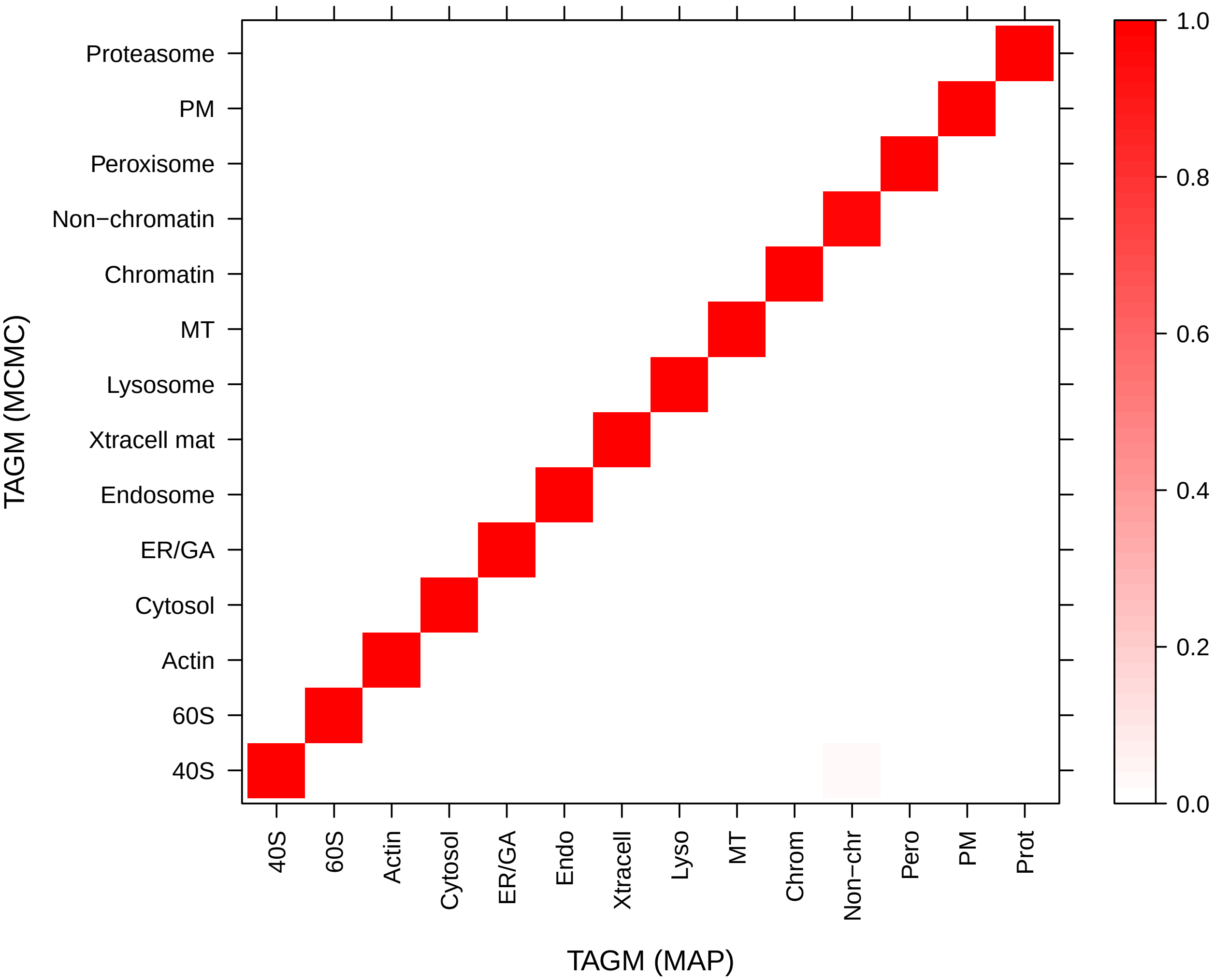

Figure D: A heatmap representation of a contingency table comparing allocation produced by MCMC and MAP methods with posterior probability threshold set at 0.99 for both methods. The scale ranges from 0 to 1 with values indicating the proportion of assigned proteins to that sub-cellular location. Values along the diagonal represent agreement between classifiers whilst other values represent disagreement. The allocations of proteins by both methods are in strong agreement.

References

Fraley, C. et al. (2005). Bayesian regularization for normal mixture estimation and model-based clustering. Technical report, Washington Univ Seattle Dept of Statistics.

Murphy, K. P. (2007). Conjugate bayesian analysis of the gaussian distribution. *Techincal Report*, **1**, 16.
